# Year-by-Year Blood Pressure Variability From Midlife to Death and Lifetime Dementia Risk

**DOI:** 10.1001/jamanetworkopen.2023.40249

**Published:** 2023-10-30

**Authors:** Melina G. H. E. den Brok, Jan Willem van Dalen, Zachary A. Marcum, Wim B. Busschers, Tessa van Middelaar, Nina Hilkens, Catharina J. M. Klijn, Eric P. Moll van Charante, Willem A. van Gool, Paul K. Crane, Eric B. Larson, Edo Richard

**Affiliations:** 1 Donders Institute for Brain, Cognition, and Behaviour, Department of Neurology, Radboud University Medical Center, Nijmegen, the Netherlands; 2 Department of Neurology, Amsterdam University Medical Center, Location AMC, Amsterdam, the Netherlands; 3 Department of General Practice, Amsterdam University Medical Center, Location AMC, Amsterdam, the Netherlands; 4 Department of Public and Occupational Health, Amsterdam University Medical Center, Location AMC, Amsterdam, the Netherlands; 5 School of Pharmacy, University of Washington, Seattle; 6 School of Medicine, University of Washington, Seattle; 7 Kaiser Permanente Washington Health Research Institute Seattle, Seattle

## Abstract

**Importance:**

High visit-to-visit blood pressure variability (BPV) in late life may reflect increased dementia risk better than mean systolic blood pressure (SBP). Evidence from midlife to late life could be crucial to understanding this association.

**Objective:**

To determine whether visit-to-visit BPV at different ages was differentially associated with lifetime incident dementia risk in community-dwelling individuals.

**Design, Setting, and Participants:**

This cohort study analyzed data from the Adult Changes in Thought (ACT) study, an ongoing population-based prospective cohort study in the US. Participants were 65 years or older at enrollment, community-dwelling, and without dementia. The study focused on a subset of deceased participants with brain autopsy data and whose midlife to late-life blood pressure data were obtained from Kaiser Permanente Washington medical archives and collected as part of the postmortem brain donation program. In the ACT study, participants underwent biennial medical assessments, including cognitive screening. Data were collected from 1994 (ACT study enrollment) through November 2019 (data set freeze). Data analysis was performed between March 2020 and September 2023.

**Exposures:**

Visit-by-visit BPV at ages 60, 70, 80, and 90 years, calculated using the coefficient of variation of year-by-year SBP measurements over the preceding 10 years.

**Main Outcomes and Measures:**

All-cause dementia, which was adjudicated by a multidisciplinary outcome adjudication committee.

**Results:**

A total of 820 participants (mean [SD] age at enrollment, 77.0 [6.7] years) were analyzed and included 476 females (58.0%). A mean (SD) of 28.4 (8.4) yearly SBP measurements were available over 31.5 (9.0) years. The mean (SD) follow-up time was 32.2 (9.1) years in 27 885 person-years from midlife to death. Of the participants, 372 (45.4%) developed dementia. The number of participants who were alive without dementia and had available data for analysis ranged from 280 of those aged 90 years to 702 of those aged 70 years. Higher BPV was not associated with higher lifetime dementia risk at age 60, 70, or 80 years. At age 90 years, BPV was associated with 35% higher dementia risk (hazard ratio [HR], 1.35; 95% CI, 1.02-1.79). Meta-regression of HRs calculated separately for each age (60-90 years) indicated that associations of high BPV with higher dementia risk were present only at older ages, whereas the association of SBP with dementia gradually shifted direction linearly from being incrementally to inversely associated with older ages.

**Conclusions and Relevance:**

In this cohort study, high BPV indicated increased lifetime dementia risk in late life but not in midlife. This result suggests that high BPV may indicate increased dementia risk in older age but might be less viable as a midlife dementia prevention target.

## Key Points

**Question:**

Is systolic blood pressure variability (BPV) at different ages during life differentially associated with lifetime dementia risk?

**Findings:**

In this cohort study of 820 participants in the Adult Changes in Thought longitudinal study, year-by-year BPV calculated over the preceding 10 years was associated with a 35% higher dementia risk only in individuals aged 90 years but not in younger persons.

**Meaning:**

Findings of this study suggest that high BPV can indicate increased incident dementia risk in older age but is less viable as a potential midlife dementia prevention target.

## Teaser

This cohort study examines the association of systolic blood pressure (BP) variability with dementia risk from age 50 years to death in participants in the Adult Changes in Thought study.

## Introduction

Midlife hypertension is associated with higher dementia risk.^1,2,3^ Blood pressure (BP) lowering may decrease dementia risk.^4,5^ However, the association between BP and dementia remains poorly understood. With older age, this association seems to dissipate or even reverse, and individuals with hypertension have lower dementia risk.^6^ Investigating BP fluctuation may help elucidate this association.^7^

High visit-to-visit BP variability (BPV), with months to years between consecutive measurements, has been associated with increased risk of cardiovascular disease and mortality.^8,9,10^ Studies that assessed the association between BPV and dementia were heterogeneous and mostly considered late-life BPV.^11,12,13,14^ Studies that investigated midlife to late-life BPV had inconsistent results.^13,15,16^ Whether these associations are age dependent, such as for mean BP, is unknown.^6^ A recent meta-analysis including predominantly older populations suggested that high BPV may be a bigger factor in dementia risk than high BP itself.^17^ Since the association between hypertension and dementia risk apparently diminishes with age, this finding may be age dependent, with BPV becoming important in later life.

Several other factors require consideration to understand the inconsistent and possibly age-dependent association. First, younger populations require protracted follow-up to investigate dementia risk because dementia mainly occurs in older age. Second, competing risk of death may be crucial because individuals with high BPV may not survive until the age ranges wherein dementia commonly manifests. Third, the underlying mechanism is unknown and might reflect reverse causality. For example, higher BPV that reflects extensive vascular damage or neurodegeneration interfering with autonomic control will be associated with short-term dementia, especially in late life.

In this study, we aimed to ascertain whether visit-to-visit BPV at different ages from 50 to 90 years was differentially associated with lifetime incident dementia risk in community-dwelling individuals who were monitored from midlife to death. Moreover, we compared these associations with those for mean BP at the same ages and examined the potential implications of competing risk of death and follow-up duration.

## Methods

We analyzed participants in the Adult Changes in Thought (ACT) study, an ongoing population-based prospective cohort study focusing on brain aging and incident dementia in the US. The University of Washington and Kaiser Permanente Washington (KPW; formerly Group Health Cooperative) Institutional Review Boards approved the present cohort study. All participants provided written informed consent. We followed the Strengthening the Reporting of Observational Studies in Epidemiology (STROBE) reporting guideline.^18^

### Population

In the ACT study, community-dwelling individuals without dementia aged 65 years or older at enrollment were randomly sampled from KPW, an integrated health care system in the Seattle, Washington, area. Individuals were enrolled in 3 stages: from 1994 to 1996 (2581), from 2000 to 2002 (811), and continuously (approximately 2000) to compensate for attrition since 2004. Participants underwent medical assessment at baseline and subsequent visits every 2 years.^19^ Race and ethnicity were self-reported (Asian; Black; White; and other, including American Indian or Alaska Native, Native Hawaiian or Pacific Islander, and other such as mixed race), and these data were collected and summarized to provide information on the potential generalizability of the results to other populations.

In the present study, we included ACT study participants who underwent brain autopsy after death as part of the brain donation program; these participants provided consent for the autopsy during the study. Of these participants specifically, detailed data on BP and medical history from midlife to late life were retrospectively collected from the KPW medical archives, complementing the ACT study’s prospective data. To ensure high data quality, extensively trained research staff performed this task manually, following a strict protocol and holding regular supervision meetings (eMethods 1 in Supplement 1). Data for the current study were collected from 1994 (ACT study enrollment) through November 2019 (data set freeze).

### BPV

We included 1 systolic BP (SBP) measurement per participant per year from age 50 years based on KPW records. Age was based on birth year and not birth date. Systolic BP measurements were examinations during consultations that followed standard clinical practice. At KPW, BP is routinely measured and recorded at medical visits, typically at least once annually, making year-by-year BP data readily available. For each participant, up to 3 measurements could be available per calendar year, 1 per block from January to April, May to August, and September to December. Having many measurements per year might indicate frequent clinical visits and thereby possibly more frequent or severe disease. To ensure a homogeneous exposure definition, if multiple measurements were recorded within a calendar year, we used only 1 (the earliest) that approximated year-by-year BP measurements.

Visit-to-visit BPV was calculated using the SBP’s coefficient of variation: (SD/mean) × 100. This measure accounts for variability increasing with higher SBP and is most commonly used, optimizing comparability of this study with previous studies.^17^ Visit-to-visit BPV was calculated per age-decade (or age by decade, such as 50-59 years) for each participant. Individuals with fewer than 3 SBP measurements available within an age-decade were excluded from analyses concerning that decade.

### Dementia

At each ACT study visit, the Cognitive Abilities Screening Instrument was administered.^20^ Participants scoring lower than 86 points underwent full dementia evaluation, including physical and neurological examinations; neuropsychological testing; and review of medical records, available laboratory testing, and imaging results. An outcome adjudication committee, including physicians, a neuropsychologist, and a research nurse, reviewed results. Dementia was diagnosed using the *Diagnostic and Statistical Manual of Mental Disorders* (Fourth Edition).^21^ Participants with dementia were followed up for 1 year after initial diagnosis to verify their diagnosis. Details of the dementia diagnostic procedure and follow-up are described elsewhere.^22^

### Covariates

For stroke, myocardial infarction (MI), and diabetes, the age at first diagnosis was derived from KPW records, and the self-reported age of first occurrence was obtained from the ACT study data set. Years of education, *APOE* genotype, smoking status, and age of smoking cessation were collected at baseline of the ACT study.

### Statistical Analysis

We performed Cox proportional hazards regression, with standardized visit-to-visit BPV over age-decades (50-59, 60-69, 70-79, and 80-89 years) as the risk factor in separate models. Censoring age was used as a timescale, with ACT study baseline age as the time of entry because participants were without dementia at baseline. The association between BPV and age at dementia diagnosis was the primary interest, and the instantaneous failure rate did not depend on enrollment date; hence, patients entered the risk set at the study inclusion age. For each analysis, we included only individuals without dementia who survived without dementia throughout the BPV period. For example, the association for BPV from 60 to 69 years reflected the estimated hazard ratio (HR) for individuals without dementia aged 70 years during their remaining lifetime according to their BPV over the preceding decade (eFigure in Supplement 1).

Model 1 was adjusted for sex and mean SBP. Model 2 also was adjusted for years of education; smoking status; *APOE* genotype (0 vs ≥1 ε4 allele); and history of stroke, MI, and diabetes as risk factors for dementia and/or elevated BPV.^1,23^ Stroke, MI, diabetes, and smoking status were coded categorically per BPV period as follows: absent (reference), new (first occurrence during BPV period), or prevalent (first occurrence before BPV period). Individuals with missing values were left out per analysis (ie, if they had missing values in variables relevant to that analysis). Proportional hazard assumptions were checked using Schoenfeld residuals.

Competing risk of death was evaluated using a cause-specific hazard approach, repeating analyses for the outcomes of mortality and dementia and mortality combined. This approach was appropriate because we aimed to study the etiological association between BPV and dementia risk and not to estimate cumulative dementia incidence (which would justify a subdistribution hazard approach, such as the Fine-Gray model).^24^

To compare associations for BPV with associations for mean SBP, we repeated the analyses with mean SBP per age-decade as the risk factor, adjusting for BPV. Subgroup analyses were performed for antihypertensive medication (AHM) use (ever vs never during life) because AHM may affect BPV^25^ and for SBP (above or below median) because low SBP may precede dementia and affect BPV.^6,26^

We conducted several sensitivity analyses. First, to investigate potential survivor biases in individuals with data available only at older age, we repeated the main analyses and excluded participants without BPV available from 50 to 59 years. Second, to examine reverse causality, which is more likely especially with short-term associations, we repeated the analyses that were stratified according to follow-up time (both <5 years and ≥5 years, and split by median time to dementia). Third, to investigate whether cardiovascular events altered the associations between BPV and dementia, we repeated the analyses and excluded individuals who ever experienced stroke or MI during their lifetime. Fourth, to evaluate whether selection of autopsy participants had implications for results, we repeated the analyses, accounting for differences between the analytic sample and the full ACT study cohort using inverse probability weighting (eMethods 2 in Supplement 1).^27^ Fifth, to investigate whether delay between the index age and ACT study baseline affected results, which it should not because we used the ACT study baseline age as time of entry, we repeated the analyses and included only individuals who had attended the ACT study baseline measurement at or before the index age. Sixth, we repeated the analyses and calculated the 10-year BPV using all available BP measurements per individual per calendar year (ie, up to 3), adjusting for the number of measurements used to calculate that 10-year BPV. All subgroup and sensitivity analyses were performed with model 2.

To further examine the potential age dependency of the associations of BPV and SBP with incident dementia, we calculated HRs for BPV and mean SBP separately over the preceding decade at each age from 60 to 90 years, as we calculated similarly for ages 60, 70, 80, and 90 years in the main analyses. We then performed a meta-regression of HRs on age using an empirical bayesian mixed-effects model^28^ and inversely weighted HRs by their SEs. The meta-regression fitted a linear or nonlinear association using natural splines with 2 degrees of freedom (1 knot at the 50th percentile), whichever fit best according to the Akaike information criterion. We chose 2 degrees of freedom to investigate nonlinear associations, but the change in association with age would unlikely be tortuous.

Two-sided *P* < .05 indicated statistical significance. Data analysis was performed between March 2020 and September 2023 using the R version 4.0.5 packages survival and meta^28,29^ (R Foundation for Statistical Computing).

## Results

**Figure 1.**
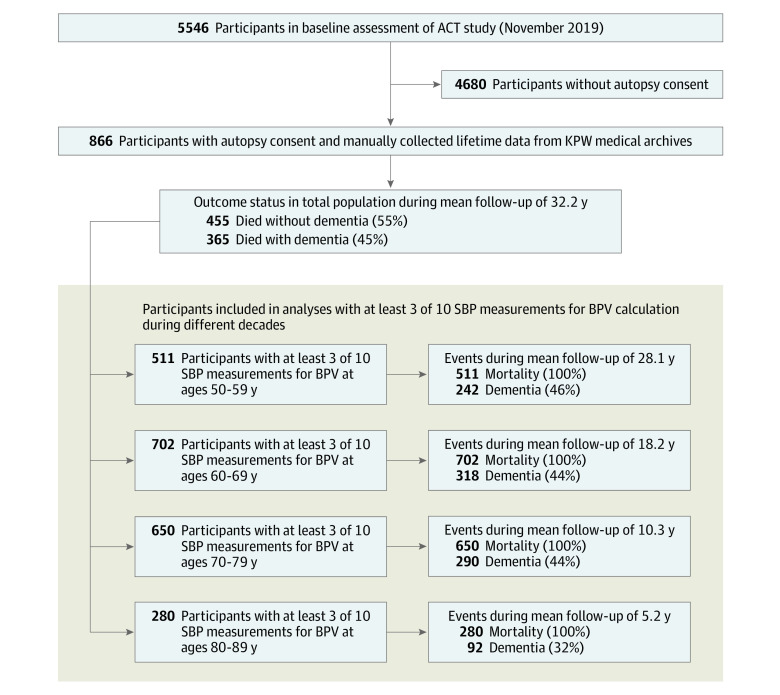
Participant Flowchart ACT indicates Adult Changes in Thought; BPV, blood pressure variability; KPW, Kaiser Permanente Washington; and SBP, systolic blood pressure.

We included 820 individuals (Figure 1), of whom 344 (42.0%) were males and 476 (58.0%) were females, with a mean (SD) age at enrollment of 77.0 (6.7) years. A mean (SD) of 28.4 (8.4) yearly SBP measurements were available over 31.5 (9.0) years. The mean (SD) follow-up time was 32.2 (9.1) years in 27 885 person-years from midlife to death.

All-cause dementia was diagnosed in 372 participants (45.4%). The number of individuals alive without dementia included per BPV period ranged from 280 of those aged 90 years to 702 of those aged 70 years (Table 1). Mean (SD) SBP increased with age from 131 (13.5) mm Hg in those aged 60 years to 141 (13.3) mm Hg in those aged 90 years, and mean (SD) BPV increased from 9.31 (3.93) in those aged 60 years to 11.22 (3.25) in those aged 90 years. Future lifetime dementia incidence ranged from 32.1% (90 of 280) in the 80 to 89 age-decade to 46.2% (221 of 478) in the 50 to 59 age-decade (Table 2).

**Table 1.** Characteristics of Individuals per Age Category

Characteristic	Index age in BPV period, No. (%)^a^			
	60 y (n = 511)	70 y (n = 702)	80 y (n = 650)	90 y (n = 280)
Sex				
Female	301 (58.9)	409 (58.3)	383 (58.9)	182 (65.0)
Male	210 (41.1)	293 (41.7)	267 (41.1)	98 (35.0)
Race and ethnicity^b^				
Asian	7 (1.4)	9 (1.3)	7 (1.1)	1 (0.4)
Black	8 (1.6)	10 (1.4)	8 (1.2)	4 (1.4)
White	475 (93.0)	662 (94.3)	614 (94.5)	268 (95.7)
Other^c^	21 (4.1)	21 (3.0)	21 (3.2)	7 (2.5)
Years of education, mean (SD), y	14.8 (3.1)	14.6 (3.0)	14.5 (3.1)	14.6 (3.1)
*APOE* genotype with ≥1 ε4 allele	144 (29.4)	184 (27.4)	158 (25.2)	55 (20.2)
Missing data	21 (41.1)	30 (4.3)	23 (3.5)	12 (4.3)
SBP per 10 y, mean (SD), mm Hg	131 (13.5)	136 (13.7)	140 (13.3)	141 (13.3)
BPV per 10 y, CV mean (SD)	9.31 (3.93)	9.67 (3.16)	10.25 (3.20)	11.22 (3.25)
Ever use of AHM				
No	446 (87.3)	522 (74.4)	377 (58.0)	109 (38.9)
Yes	65 (12.7)	180 (25.6)	273 (42.0)	171 (61.1)
Smoking status				
No	449 (90.0)	628 (91.4)	601 (93.8)	270 (97.8)
Quit during BPV period	7 (1.4)	12 (1.7)	11 (1.7)	0
Current smoker	43 (8.6)	47 (6.8)	29 (4.5)	6 (2.2)
Missing data	12 (2.3)	15 (2.1)	12 (1.8)	10 (4.3)
Myocardial infarction				
No	491 (96.1)	637 (90.7)	523 (80.5)	188 (67.1)
New event during BPV period	15 (2.9)	39 (5.6)	73 (11.2)	46 (16.4)
Yes, preceding BPV period	5 (1.0)	26 (3.7)	54 (8.3)	46 (16.4)
Diabetes				
No	483 (94.5)	633 (90.2)	567 (87.2)	243 (86.8)
Diagnosed during BPV period	20 (3.9)	36 (5.1)	30 (4.6)	21 (7.5)
Yes, preceding BPV period	8 (1.6)	33 (4.7)	53 (8.2)	16 (5.7)
Stroke				
No	502 (98.2)	651 (92.7)	533 (82.0)	189 (67.5)
New event during BPV period	6 (1.2)	37 (5.3)	79 (12.2)	51 (18.2)
Yes, preceding BPV period	3 (0.6)	14 (2.0)	38 (5.8)	40 (14.3)

Abbreviations: ACT, Adult Changes in Thought; AHM, antihypertensive medication; BPV, blood pressure variability; CV, coefficient of variation; SBP, systolic blood pressure.

^a^ Individuals can be included in multiple columns since they were monitored for several decades from the moment of enrollment in the ACT study until death or dementia diagnosis. From the 820 participants included in the study, only the characteristics of individuals with available BPV measurements are depicted.

^b^ Race and ethnicity were self-reported.

^c^ Other category included American Indian or Alaska Native, Native Hawaiian or Pacific Islander, and other, such as mixed race.

**Table 2.** Hazard Ratios (HRs) for Incident Dementia and Mortality per 1-SD Increment in Blood Pressure Variability (BPV)^a^

Age, y	BPV period, age-decade, y	No./Total No. of events (%)		HR (95% CI)^b^		
		Dementia	Mortality	Dementia	Mortality	Dementia and mortality combined
**Model 1** ^c^						
60	50-59	236/511 (46.2)	511/511 (100)	0.99 (0.87-1.13)	1.03 (0.94-1.13)	1.02 (0.94-1.12)
70	60-69	311/702 (44.3)	703/703 (100)	0.91 (0.81-1.02)	1.02 (0.95-1.10)	0.97 (0.90-1.05)
80	70-79	285/650 (43.8)	693/693 (100)	1.01 (0.90-1.15)	1.17 (1.08-1.27)	1.12 (1.03-1.21)
90	80-89	90/280 (32.1)	394/394 (100)	1.27 (0.98-1.65)	1.22 (1.08-1.38)	1.23 (1.06-1.44)
**Model 2** ^d^						
60	50-59	221/478 (46.2)	478/478 (100)	0.97 (0.85-1.11)	1.04 (0.95-1.14)	1.02 (0.93-1.12)
70	60-69	290/657 (44.1)	658/658 (100)	0.91 (0.80-1.02)	1.01 (0.93-1.09)	0.96 (0.88-1.03)
80	70-79	271/619 (43.8)	659/659 (100)	1.02 (0.89-1.16)	1.14 (1.05-1.24)	1.09 (1.00-1.19)
90	80-89	85/268 (31.7)	373/373 (100)	1.35 (1.02-1.79)	1.16 (1.02-1.32)	1.19 (1.01-1.41)

^a^ Cox proportional hazards regression was performed for individuals at ages 60, 70, 80, and 90 years who were alive without dementia according to BPV calculated over the preceding 10 years (BPV period).

^b^ Calculated for lifetime risks.

^c^ Model 1 adjusted for sex and mean systolic blood pressure.

^d^ Model 2 also adjusted for sex and mean systolic blood pressure; years of education; smoking status; *APOE* genotype; and history of stroke, myocardial infarction, and diabetes.

### Main Analyses

Table 2 shows results of the fully adjusted model 2. Higher BPV was not associated with higher dementia risk at ages 60 (HR, 0.97; 95% CI, 0.85-1.11), 70 (HR, 0.91; 95% CI, 0.80-1.02), and 80 (HR, 1.02; 95% CI, 0.89-1.16) years. At age 90 years, a 1-SD increment in BPV was associated with a 35% higher dementia risk (HR, 1.35; 95% CI, 1.02-1.79). For mortality, associations were neutral at ages 60 (HR, 1.04; 95% CI, 0.95-1.14) and 70 (HR, 1.01; 95% CI, 0.93-1.09) years. At ages 80 (HR, 1.14; 95% CI, 1.05-1.24) and 90 (HR, 1.16; 95% CI, 1.02-1.32) years, a 1-SD increment in BPV indicated a 14% to 16% higher mortality risk, respectively. Associations with dementia and mortality combined were neutral at ages 60 (HR, 1.02; 95% CI, 0.93-1.12) and 70 (HR, 0.96; 95% CI, 0.88-1.03) years. At ages 80 (HR, 1.09; 95% CI, 1.00-1.19) and 90 (HR, 1.19; 95% CI, 1.01-1.41) years, a 1-SD increment in BPV indicated a 9% and 19% higher risk, respectively.

Analyses for mean SBP showed no associations (Table 3). However, a gradual shift in direction of associations was apparent with older age: from 7% higher dementia risk per SD at age 60 years (HR, 1.07; 95% CI, 0.93-1.23) to a neutral risk at age 70 years (HR, 0.99; 95% CI, 0.87-1.11), 10% lower risk at age 80 years (HR, 0.90; 95% CI, 0.79-1.01), and 18% lower risk at age 90 years (HR, 0.82; 95% CI, 0.65-1.03). Unadjusted results were similar for BPV. Systolic BP was not associated with mortality (Table 3).

**Table 3.** Hazard Ratios (HRs) for Incident Dementia and Mortality per 1-SD Increment in Mean Systolic Blood Pressure (SBP)^a^

Age, y	SBP period, age-decade, y	No./Total No. of events (%)		HR (95% CI)^b^		
		Dementia	Mortality	Dementia	Mortality	Dementia and mortality combined
**Model 1** ^c^						
60	50-59	221/478 (46.2)	478/478 (100)	1.10 (0.96-1.26)	1.02 (0.92-1.12)	1.02 (0.93-1.12)
70	60-69	290/657 (44.1)	657/657 (100)	1.01 (0.90-1.14)	1.01 (0.93-1.09)	1.01 (0.94-1.09)
80	70-79	271/619 (43.8)	619/619 (100)	0.94 (0.83-1.05)	0.97 (0.89-1.05)	0.95 (0.88-1.03)
90	80-89	85/268 (31.7)	268/268 (100)	0.86 (0.69-1.08)	0.92 (0.81-1.05)	0.90 (0.79-1.02)
**Model 2** ^d^						
60	50-59	221/478 (46.2)	478/478 (100)	1.07 (0.93-1.23)	1.01 (0.91-1.11)	1.00 (0.91-1.11)
70	60-69	290/657 (44.1)	657/657 (100)	0.99 (0.87-1.11)	0.99 (0.91-1.07)	0.99 (0.92-1.08)
80	70-79	271/619 (43.8)	619/619 (100)	0.90 (0.79-1.01)	0.96 (0.88-1.04)	0.93 (0.86-1.01)
90	80-89	85/268 (31.7)	268/268 (100)	0.82 (0.65-1.03)	0.96 (0.84-1.10)	0.91 (0.79-1.03)

^a^ Cox proportional hazards regression was performed for individuals at ages 60, 70, 80, and 90 years who were alive without dementia according to mean SBP calculated over the preceding 10 years (SBP period).

^b^ Calculated for lifetime risks.

^c^ Model 1 adjusted for sex and mean SBP.

^d^ Model 2 also adjusted for sex and mean SBP; years of education; smoking status; *APOE* genotype; and history of stroke, myocardial infarction, and diabetes.

### Subgroup and Sensitivity Analyses

Results between subgroups according to AHM use (eTable 1 in Supplement 1) were not significantly different. Analyses that were stratified according to median SBP showed no significant differences (eTable 2 in Supplement 1).

Sensitivity analyses excluding individuals without BPV available at age 60 years from all analyses yielded similar results (eTable 3 in Supplement 1). Analyses according to follow-up time showed no clear differences between less than 5 years vs 5 years or more of follow-up (eTable 4 in Supplement 1). Regarding a split according to the median time to dementia (eTable 5 in Supplement 1), dementia HRs for BPV at age 90 years were higher in the long term (>3.5 years: HR, 1.52; 95% CI, 1.07-2.16) than the short term (HR, 1.15; 95% CI, 0.79-1.68). Analyses excluding individuals with lifetime stroke (eTable 6 in Supplement 1) and lifetime MI (eTable 7 in Supplement 1) showed results similar to the main analyses, accounting for autopsy cohort selection (eTable 8 in Supplement 1) and only including individuals with ACT study baseline measurement at or before the index age (eTable 9 in Supplement 1). Analyses using all available SBP measurements per individual to calculate 10-year BPV (eTable 10 in Supplement 1) showed slightly attenuated results.

**Figure 2.**
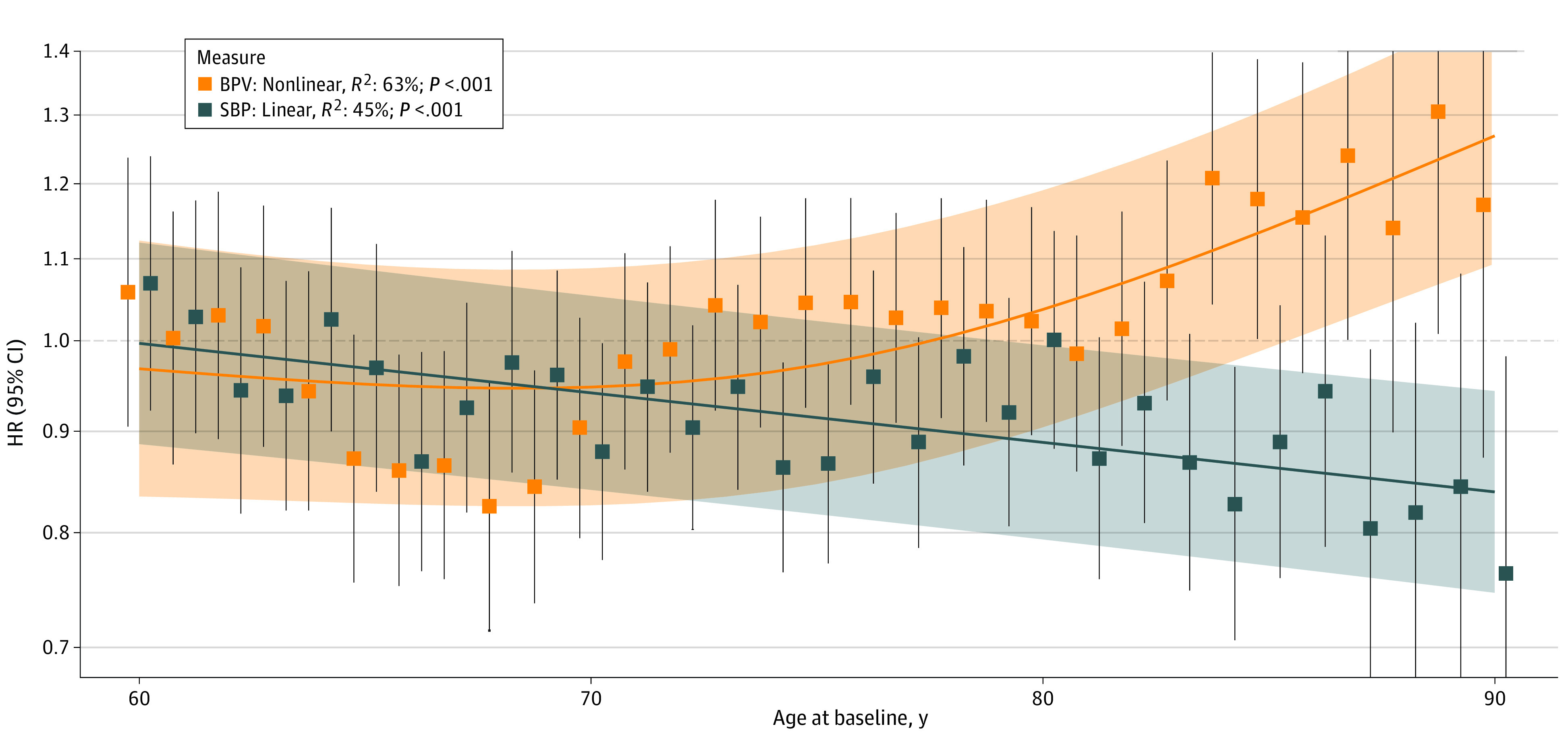
Hazard Ratios (HRs) for Association of Blood Pressure Variability (BPV) and Systolic Blood Pressure (SBP) With Dementia at Different Ages From 60 to 90 Years Depicted are best-fitting regression lines: nonlinear for BPV and linear for SBP, with shaded areas representing credible intervals. *R*^2^ values represent the percentages of heterogeneity between the HRs at different ages explained by each regression line. Change in association between BPV and dementia risk with older age best fit a nonlinear vs linear model (Akaike information criterion [AIC], −72.9 vs −64.3). Change in association between SBP and dementia risk with older age best fit a linear vs nonlinear model (AIC, −84.2 vs −85.9).

Figure 2 presents HRs of dementia for BPV and mean SBP at each age from 60 to 90 years. Meta-regression suggested that the association of BPV with elevated dementia risk only developed nonlinearly with older age. The meta-regression line explained 63% of heterogeneity between HRs. The difference in association between SBP and dementia risk with older age was inversely linear, with meta-regression explaining 45% of heterogeneity between HRs.

## Discussion

In this cohort study of 820 community-dwelling individuals monitored for a mean time of 32.2 years, only higher BPV in older age was associated with higher dementia risk. This finding could not be explained by opposite association for mortality as a competing risk. At age 90 years, HRs seemed higher for a longer time to dementia. Results did not significantly differ between subgroups according to SBP or AHM use and were similar when individuals with lifetime stroke or MI were excluded. Meta-regression corroborated that the association of high BPV with higher dementia risk developed only at older age, whereas the direction of associations for SBP with dementia risk shifted from incrementally associated to inversely associated with older age.

To our knowledge, no previous studies have examined BPV spanning over 30 years from midlife to death within the same individuals, making the current study uniquely suited to examine the role that age plays in the association between BPV and dementia. Higher BPV mainly indicated higher dementia risk at older ages. A recent meta-analysis reported 27% higher odds (odds ratio, 1.27; 95% CI, 1.17-1.38) of an association between elevated systolic visit-to-visit BPV and incident dementia or cognitive impairment.^17^ Nine of the 10 studies in this meta-analysis examined older populations (mean age >65 years). Associations with cognitive impairment might emerge earlier because it may precede dementia by several years.^30^ The only study of individuals in midlife (mean age, 54 years) found that individuals with high BPV had lower cognition, but no association with subsequent cognitive decline over 15 years was observed.^16^ Studies with subgroup analyses according to age had heterogeneous findings.^14,15^ Two of these studies observed the highest dementia risk with elevated BPV in participants younger than 65 years and younger than 70 years,^13,15^ and 1 study reported similar findings in participants 70 years or older.^14^ None investigated age-specific associations for BPV with lifetime dementia risk within the same individuals. Therefore, generational biases and older age–related inclusion bias may have affected these results. Moreover, median follow-up ranged from 6 to 15 years, and thus outcomes may have been missed in younger people since dementia generally occurs later than age 80 years.^31^

The change in association between SBP and dementia risk from incrementally to inversely associated with older age matches findings in the literature, in which midlife hypertension indicated higher dementia risk^1^ and late-life hypertension indicated lower risk.^6,32,33^ Results of the present study support suggestions in a meta-analysis^17^ that BPV might be a bigger factor in dementia risk than SBP, but this association seemed specific for BPV at older age.

The mechanism in the association between BPV and dementia is unknown.^7^ The finding in this study that midlife BPV did not affect lifetime dementia risk makes a causal, cumulative dose-response association less likely. Causality might be reversed, with high BPV reflecting the implications of neurodegeneration for the autonomic nervous system^34^ or extensive vascular damage. The association of BPV at 90 years with dementia was particularly notable in the longer term (>3.5 years), contesting the theory that high BPV prodromally preceded dementia. Combined with findings that high BPV was a risk factor for cerebral small vessel disease^35^ and vascular outcomes, including chronic kidney disease,^36^ MI, and stroke,^8,9,37^ an association with cerebrovascular disease seemed plausible. Alternatively, the body’s ability to maintain homeostasis may decrease with aging, making older age–related elevated BPV a marker of advanced biological age and thereby associated with dementia. Variability in other cardiovascular, metabolic, and kidney parameters in older people has also been associated with increased risk of cardiovascular events and dementia.^38,39,40^ Excluding individuals with lifetime stroke and subgroup analyses for AHM use yielded largely unaltered results, suggesting that the association between BPV and dementia is not solely dependent on poststroke dementia or AHM use.

### Strengths and Limitations

Strengths of this study are the year-by-year BP data available for the same community-dwelling individuals for over 30 years from midlife to death, along with systematic biennial cognitive assessment and dementia outcome confirmation by an adjudication committee. Because they originated from the same individuals, data at different ages more closely represented aging-related outcomes rather than potential age-related or generational selection biases. The long follow-up time allowed for analyzing lifetime dementia risk, even for BPV at younger ages, wherein limited follow-up duration usually necessitates analyzing cognitive change scores as outcomes. Using dementia diagnoses as an outcome was critical because cognitive decline may often not result in dementia.^30^

This study also has limitations. First, we included only the autopsy data of consenting ACT study participants. The high dementia rate in this sample (45.4%) may partly reflect these individuals’ increased willingness to consent to autopsy along with the expected lifetime dementia risk for individuals 65 years or older being 30% to 40%,^41^ and the biennial cognitive screening identifying extra cases. These factors may restrict the generalizability of the findings. However, the sensitivity analyses that accounted for differences between the autopsy sample and the full ACT study population yielded similar results, suggesting that the results are generalizable to the ACT study population. However, the study population included mostly White individuals in the US with access to relatively high-quality health care, possibly limiting the generalizability of the findings to more diverse samples and/or regions with less access to health care. Second, the ACT study’s enrollment of individuals 65 years or older might have selected survivors who were relatively impervious to high BPV. Third, variance in methods of SBP assessors may have played a role in higher BPV estimates. However, this divergence is unlikely to systematically differ between individuals with vs without incident dementia. Although 1 to 3 measurements per year were available per participant, we included only 1 to homogenize exposure operationalization across individuals. Sensitivity analyses that included all available SBP measurements showed slightly attenuated associations. This finding may reflect the differences in exposure definition per participant and the number of measurements that possibly depend on health status, distorting the associations. Alternatively, association for year-by-year BPV may differ from BPV in shorter periods.^42^ Fourth, we performed subgroup analyses only comparing ever vs never AHM users, but analyzing AHM classes and (cumulative) doses may be warranted since these factors might affect BPV.

## Conclusion

In this cohort study, high BPV in late life, but not in midlife, was associated with higher lifetime dementia risk. This finding was not due to excess mortality. Results suggest that high BPV may indicate increased dementia risk in older age but might be less viable as a midlife dementia prevention target. This study was unique in assessing the associations of BPV with lifetime dementia risk at multiple ages from midlife to late life, underlining the necessity of additional long-term studies with more diverse samples to confirm the age dependency of this association, identify underlying mechanisms, and corroborate this study’s conclusions.

## Notes

Supplement 1. **eMethods 1.** ACT Chart Review Process **eMethods 2.** Weighted Sensitivity Analysis Evaluating Potential Cohort Selection Effects **eTable 1.** Hazard Ratios per One Standard Deviation Increment in Systolic BPV at Ages 60, 70, 80, and 90 for Incident Dementia in Antihypertensive Medication Users and Non-Users **eTable 2.** Hazard Ratios per One Standard Deviation Increment in Systolic BPV at Ages 60, 70, 80, and 90 for Incident Dementia Subgroups Stratified According to Median Systolic Blood Pressure **eTable 3.** Hazard Ratios per One Standard Deviation Increment in Systolic BPV at Ages 60, 70, 80, and 90 for Incident Dementia Excluding Individuals Without BPV Measurement Available at Age 60 **eTable 4.** Hazard Ratios per One Standard Deviation Increment in Systolic BPV at Ages 60, 70, 80, and 90 for Incident Dementia According to Moment of Censoring (<5 or ≥5 Years of Follow-Up) **eTable 5.** Hazard Ratios per One Standard Deviation Increment in Systolic BPV at Ages 60, 70, 80, and 90 for Incident Dementia According to Median to Time to Dementia Diagnosis **eTable 6.** Hazard Ratios per One Standard Deviation Increment in Systolic BPV at Ages 60, 70, 80, and 90 for Incident Dementia, Excluding Individuals With Stroke at Any Age During Life **eTable 7.** Association for One SD Increase in Mean Systolic Blood Pressure Variability Over Previous 10-Years and Lifetime Dementia or Mortality Risk, Excluding Individuals With Myocardial Infarction at Any Age During Life **eTable 8.** Association for One SD Increase in Mean Systolic Blood Pressure Variability Over Previous 10-Years and Lifetime Dementia or Mortality Risk, Using Inverse Probability Weighting to Account for Potential Selection Into the Autopsy Cohort From the Larger ACT Study Population **eTable 9.** Association for One SD Increase in Mean Systolic Blood Pressure Variability Over Previous 10-Years and Lifetime Dementia or Mortality Risk, Only Including Individuals Who Had Attended ACT Baseline At or Before the Index Age **eTable 10.** Association for One SD Increase in Mean Systolic Blood Pressure Variability Over Previous 10-Years and Lifetime Dementia or Mortality Risk, Using 10-Year BPV Calculated With All Available SBP Measurements per Individual per Year (Maximum 3) Adjusted for the Number of SBP Measurements **eFigure.** Depiction of BPV Periods in Relation to Analysis Index Age

Supplement 2.
Data Sharing Statement
