# Supplementary material for: Year-by-Year Blood Pressure Variability From Midlife to Death and Lifetime Dementia Risk
Source: JAMA Netw Open. 2023 Oct 30;6(10):e2340249. doi: 10.1001/jamanetworkopen.2023.40249 (PMC10616718; doi:10.1001/jamanetworkopen.2023.40249)
Supplement: Supplement 1. — eMethods 1. ACT Chart Review Process eMethods 2. Weighted Sensitivity Analysis Evaluating Potential Cohort Selection Effects eTable 1. Hazard Ratios per One Standard Deviation Increment in Systolic BPV at Ages 60, 70, 80, and 90 for Incident Dementia in Antihypertensive Medication Users and Non-Users eTable 2. Hazard Ratios per One Standard Deviation Increment in Systolic BPV at Ages 60, 70, 80, and 90 for Incident Dementia Subgroups Stratified According to Median Systolic Blood Pressure eTable 3. Hazard Ratios per One Standard Deviation Increment in Systolic BPV at Ages 60, 70, 80, and 90 for Incident Dementia Excluding Individuals Without BPV Measurement Available at Age 60 eTable 4. Hazard Ratios per One Standard Deviation Increment in Systolic BPV at Ages 60, 70, 80, and 90 for Incident Dementia According to Moment of Censoring (<5 or ≥5 Years of Follow-Up) eTable 5. Hazard Ratios per One Standard Deviation Increment in Systolic BPV at Ages 60, 70, 80, and 90 for Incident Dementia According to Median to Time to Dementia Diagnosis eTable 6. Hazard Ratios per One Standard Deviation Increment in Systolic BPV at Ages 60, 70, 80, and 90 for Incident Dementia, Excluding Individuals With Stroke at Any Age During Life eTable 7. Association for One SD Increase in Mean Systolic Blood Pressure Variability Over Previous 10-Years and Lifetime Dementia or Mortality Risk, Excluding Individuals With Myocardial Infarction at Any Age During Life eTable 8. Association for One SD Increase in Mean Systolic Blood Pressure Variability Over Previous 10-Years and Lifetime Dementia or Mortality Risk, Using Inverse Probability Weighting to Account for Potential Selection Into the Autopsy Cohort From the Larger ACT Study Population eTable 9. Association for One SD Increase in Mean Systolic Blood Pressure Variability Over Previous 10-Years and Lifetime Dementia or Mortality Risk, Only Including Individuals Who Had Attended ACT Baseline At or Before the Index Age eTable 10. Association for O [file jamanetwopen-e2340249-s001.pdf]

Supplemental Online Content

den Brok MGHE, van Dalen JW, Marcum ZA, et al. Year-by-year blood pressure variability from midlife to death and lifetime dementia risk. *JAMA Netw Open*. 2023;6(10):e2340249. doi:10.1001/jamanetworkopen.2023.40249

eMethods 1. ACT Chart Review Process

eMethods 2. Weighted Sensitivity Analysis Evaluating Potential Cohort Selection Effects

eTable 1. Hazard Ratios per One Standard Deviation Increment in Systolic BPV at Ages 60, 70, 80, and 90 for Incident Dementia in Antihypertensive Medication Users and Non-Users

eTable 2. Hazard Ratios per One Standard Deviation Increment in Systolic BPV at Ages 60, 70, 80, and 90 for Incident Dementia Subgroups Stratified According to Median Systolic Blood Pressure

eTable 3. Hazard Ratios per One Standard Deviation Increment in Systolic BPV at Ages 60, 70, 80, and 90 for Incident Dementia Excluding Individuals Without BPV Measurement Available at Age 60

eTable 4. Hazard Ratios per One Standard Deviation Increment in Systolic BPV at Ages 60, 70, 80, and 90 for Incident Dementia According to Moment of Censoring (<5 or ≥5 Years of Follow-Up)

eTable 5. Hazard Ratios per One Standard Deviation Increment in Systolic BPV at Ages 60, 70, 80, and 90 for Incident Dementia According to Median to Time to Dementia Diagnosis

eTable 6. Hazard Ratios per One Standard Deviation Increment in Systolic BPV at Ages 60, 70, 80, and 90 for Incident Dementia, Excluding Individuals With Stroke at Any Age During Life

eTable 7. Association for One SD Increase in Mean Systolic Blood Pressure Variability Over Previous 10-Years and Lifetime Dementia or Mortality Risk, Excluding Individuals With Myocardial Infarction at Any Age During Life

eTable 8. Association for One SD Increase in Mean Systolic Blood Pressure Variability Over Previous 10-Years and Lifetime Dementia or Mortality Risk, Using Inverse Probability Weighting to Account for Potential Selection Into the Autopsy Cohort From the Larger ACT Study Population

© 2023 den Brok MGHE et al. *JAMA Network Open*

eTable 9. Association for One SD Increase in Mean Systolic Blood Pressure Variability Over Previous 10-Years and Lifetime Dementia or Mortality Risk, Only Including Individuals Who Had Attended ACT Baseline At or Before the Index Age

eTable 10. Association for One SD Increase in Mean Systolic Blood Pressure Variability Over Previous 10-Years and Lifetime Dementia or Mortality Risk, Using 10-Year BPV Calculated With All Available SBP Measurements per Individual per Year (Maximum 3) Adjusted for the Number of SBP Measurements

eFigure. Depiction of BPV Periods in Relation to Analysis Index Age

This supplemental material has been provided by the authors to give readers additional information about their work.

eMethods 1. ACT chart review process

For the adult changes in thought (ACT) study chart review project, the prospective data recorded during the Adult Changes in Thought study, was supplemented with high-quality detailed medical data from the Kaiser Permanente Washington medical archives. Study participants provided informed consent and all study activities were approved by the Kaiser Permanente Washington Institutional Review Board. These methods describe the set-up of the chart review project, the measures taken to ensure data quality, and the collected variables.

Development and quality assurance

The initial medical record abstraction team included a physician and three medical record review professionals. The team met on a recurrent basis to discuss and outline the project. We reviewed local experience with medical record abstractions and requested materials from a research group that had published numerous case-control studies using medical records from the same healthcare delivery system.

The overall goal was to collect longitudinal medical record data across the participant's entire enrolment in the healthcare delivery system. Because of the magnitude of the task, we developed a computerized medical record abstraction tool, facilitating direct data entry into a database rather than initial abstraction onto paper forms requiring subsequent data entry.

We carefully considered data elements to abstract. We were guided by the parent study's specific aims, which at the time included specific attention to pharmacoepidemiology and in the subsequent grant cycle focused on vascular risk factors. We also considered the wide variety of data collected by the ACT study at study visits and considered whether there was likely to be additional value from abstracting related data from participant medical records. We consulted clinical researchers to review sections of the medical record abstraction in their specific area of expertise.

Documentation was a crucial component of the medical record abstraction project, and study staff prepared an extensive draft Manual of Operations to detail specific instructions about each data field. Study staff also prepared a question log in which they recorded the rationale for decisions of what to collect and what not to collect.

Due to early concerns regarding the amount of time necessary for abstracting data, the team reviewed each variable to consider its utility. The team was guided by considerations of validity and efficiency. As much as possible, we tried to facilitate instructions that would allow medical record abstractors to "abstract what you see," that is, to record what they observed in the medical records rather than trying to guess what was occurring in the mind of the provider or the body of the study participant.

Medical record abstractors abstracted data from an initial group of 40 medical records using the medical record abstraction tool. As part of quality assurance, 15 of these medical records were abstracted by more than one abstractor. The team discussed difficulties and discrepancies with each other and with frequent meetings with the lead investigator. Based on these discussions, we further modified the tool and the Manual of Operations. The team established a workflow with rapid cycles of updating the medical record abstraction tool.

Training and ongoing quality control

During the initial training period for new abstractors, all medical records abstracted by medical record abstractors were also abstracted by the lead abstractor. We required three consecutive medical records with greater than 93% agreement on selected fields with the lead abstractor to certify abstractors for this project. Fields considered for this threshold included presence/absence of medical conditions and procedures, demographics, social history, dates and numeric values for laboratory values, weights, and blood pressures. New medical record abstractors were permitted to abstract data on medical records only after completing the certification.

© 2023 den Brok MGHE et al. *JAMA Network Open*

We have established extensive ongoing quality control efforts, including an inter-rater reliability protocol with a random 5% re-abstraction, with careful attention to discrepancies, and computer-generated error prevention and detection techniques.

The team continues to meet on a routine basis with the lead investigator. Routinely scheduled meetings include team meetings without the lead investigator, meetings with the computer programmer, and meetings to discuss re-abstraction findings.

As an estimate of the time spent per patient on data extraction, including training periods and continuous quality improvement efforts, abstracting data for the first 380 medical records took 2,352 person-hours, an average of 6.2 hours per participant.

Data elements

The medical record abstraction project includes abstraction of medication data in specific categories including antidepressants, antihypertensives, antipsychotics, diabetes medications, hormones, thyroid medications, and sedatives from years before 1977, the year electronic pharmacy record became available at Kaiser Permanente Washington (formerly Group Health). Because of difficulties abstracting accurate data, the team only abstracts drug names, with no attempt to gather dosing information. Medication data prior to 1977 are coded using a pre-populated drug library. From 1977 onwards, the ceased medications drug library is generated with a participant-specific list of medications from computerized pharmacy data.

The team records blood pressures up to three times per year, divided into 4-month bins from January through April, May through August, and September through December. If there are multiple blood pressures within a four-month bin, a hierarchical scheme preferentially chooses seated, repeat blood pressure readings performed by physicians in an outpatient setting using the following scheme. The first blood pressure (BP) taken in each period per year is recorded. Priority is given to an outpatient blood pressure, even if an inpatient BP occurs earlier in the time period. If there are multiple blood pressures listed during a single visit or time period, only one is recorded according to the following criteria:

- Clinic visit BP has priority over ER/urgent care/hospital visit BP
- Right arm BP has priority over Left arm BP
- Seated BP has priority over Lying BP over Standing BP
- MD taken BP has priority over nursing staff BP
- Repeat BP has priority over initial BP during a single visit

The team abstracts tobacco use on an annual basis across all years. The team abstracts several cardiovascular conditions, including stroke and myocardial infarction. The team also abstracts a variety of non-cardiovascular medical conditions, including diabetes.

The team enters abstracted data directly into a database application that uses Microsoft Access as its front-end interface (i.e., data entry forms) and Microsoft SQL Server as the underlying database engine. Programmers employed a wide variety of data form controls to minimize errors, including options groups, checkboxes, and drop-down lists. Programmers added alerts, range, and logic checks to date and numeric fields to prevent entry of out-of-range values. There are also program scripts that selectively enable or disable data fields depending on value(s) entered in other fields. We also implemented a tool that locks down a subject's data set once it is deemed 'complete' to eliminate the chance of accidentally altering a cleaned and verified medical record abstraction.

Abstracted data are stored in a secure SQL Server database. Access is restricted to members of the medical record review team who have been specifically granted permission.

© 2023 den Brok MGHE et al. *JAMA Network Open*

eMethods 2. Weighted sensitivity analysis evaluating potential cohort selection effects

Selection bias may occur if demographic and clinical factors are associated with inclusion in the autopsy cohort (including consent to autopsy, study withdrawal, and death). To evaluate whether this had any effects on our main analyses, we performed a sensitivity analysis using inverse probability weighting to account for differences that might exist between the analytic sample and the broader ACT cohort, similar as described previously.(1) Weights were derived as the inverse of inclusion probability estimated from a logistic regression model on selection using all ACT participants. Predictors in the selection model included age at ACT study entry, ACT cohort (i.e., original first wave, second expansion wave, or the subsequently established continuous replacement cohort), sex, education, dementia, systolic blood pressure, antihypertensive use, history of stroke, and history of coronary artery disease. To account for the uncertainty in the estimated weights, we used bootstrap analyses to calculate 1,000 weights for each participant. We subsequently repeated the main analyses calculating the effect sizes and 95%CI using 10,000 bootstraps, each time using inverse probability weighting according to a random iteration of the 1,000 probability weights. Effect sizes with 95% confidence intervals were defined as the median with the 2.5th and 97.5th percentile results of those 10,000 bootstraps.

1.) Haneuse S, Schildcrout J, Crane P, Sonnen J, Breitner J, Larson E. Adjustment for Selection Bias in Observational Studies with Application to the Analysis of Autopsy Data. Neuroepidemiology 2009;32(3):229.

© 2023 den Brok MGHE et al. *JAMA Network Open*

eTable 1. Hazard ratios per one standard deviation increment in systolic BPV at ages 60, 70, 80, and 90 for incident dementia in antihypertensive medication users and non-users

		Antihypertensive medication users		Antihypertensive medication non-users		
**Age**	**BPV period**	**Total / dementia (%)**	**HR (95%CI)**	**Total number of individuals**	**HR (95%CI)**	**p-value interaction**
**60**	**50-59**	63 / 27 (42.9)	1.07 (0.72 - 1.58)	415 / 194 (46.7)	0.94 (0.81 - 1.09)	0.31
**70**	**60-69**	166 / 67 (40.4)	1.07 (0.80 - 1.43)	491 / 223 (45.4)	0.88 (0.77 - 1.01)	0.26
**80**	**70-79**	260 / 103 (39.6)	1.00 (0.78 - 1.28)	359 / 168 (46.8)	1.02 (0.86 - 1.21)	0.75
**90**	**80-89**	165 / 48 (29.1)	1.43 (0.99 - 2.06)	103 / 37 (35.9)	1.32 (0.77 - 2.26)	0.57

Cox proportional hazards for individuals at ages 60, 70, 80 and 90 years, alive without dementia, according to blood pressure variability (BPV) calculated of over the preceding 10 years (BPV period). Hazard ratios indicate lifetime risks. Adjusted for sex, mean systolic blood pressure, years of education, smoking status, ApoE genotype and history of stroke, myocardial infarction and diabetes mellitus. Abbreviations: HR= hazard ratio, CI=confidence interval, BPV=blood pressure variability, SBP: systolic blood pressure

© 2023 den Brok MGHE et al. *JAMA Network Open*

eTable 2. Hazard ratios per one standard deviation increment in systolic BPV at ages 60, 70, 80, and 90 for incident dementia subgroups stratified according to median systolic blood pressure

		< median SBP		> median SBP		
**Age**	**BPV period**	**Total / dementia events (%)**	**HR dementia (95%CI)**	**Total / dementia events (%)**	**HR dementia (95%CI)**	**p-value interaction**
**60**	**50-59**	238 / 106 (44.5)	1.09 (0.90 - 1.33)	240 / 115 (47.9)	0.90 (0.74 - 1.09)	0.26
**70**	**60-69**	324 / 139 (42.9)	0.95 (0.80 - 1.13)	333 / 151 (45.3)	0.85 (0.72 - 1.01)	0.26
**80**	**70-79**	304 / 135 (44.4)	1.01 (0.83 - 1.24)	315 / 136 (43.2)	1.02 (0.86 - 1.22)	0.72
**90**	**80-89**	132 / 43 (32.6)	1.37 (0.86 - 2.18)	136 / 42 (30.9)	1.49 (0.94 - 2.36)	0.66

Cox proportional hazards for individuals at ages 60, 70, 80 and 90 years, alive without dementia, according to blood pressure variability (BPV) calculated of over the preceding 10 years (BPV period). Hazard ratios indicate lifetime risks. Adjusted for sex, mean systolic blood pressure, years of education, smoking status, ApoE genotype and history of stroke, myocardial infarction and diabetes mellitus. Abbreviations: HR= hazard ratio, CI=confidence interval, BPV=blood pressure variability, SBP: systolic blood pressure

© 2023 den Brok MGHE et al. *JAMA Network Open*

eTable 3. Hazard ratios per one standard deviation increment in systolic BPV at ages 60, 70, 80, and 90 for incident dementia excluding individuals without BPV measurement available at age 60

Age	BPV period	Total / dementia events (%)	Total / mortality events (%)	HR dementia (95%CI)	HR mortality (95%CI)	HR dementia/ mortality (95%CI)
**60**	**50-59**	508 / 226 (44.5)	478 / 478 (100)	0.95 (0.83 - 1.09)	1.04 (0.95 - 1.14)	1.01 (0.92 - 1.11)
**70**	**60-69**	495 / 221 (44.6)	467 / 467 (100)	0.84 (0.73 - 0.97)	1.01 (0.92 - 1.11)	0.93 (0.84 - 1.02)
**80**	**70-79**	396 / 176 (44.4)	409 / 409 (100)	0.95 (0.8 - 1.11)	1.16 (1.05 - 1.29)	1.11 (0.99 - 1.23)
**90**	**80-89**	154 / 51 (33.1)	208 / 208 (100)	1.32 (0.91 - 1.91)	1.07 (0.91 - 1.25)	1.11 (0.89 - 1.37)

Cox proportional hazards for individuals at ages 60, 70, 80 and 90 years, alive without dementia, according to blood pressure variability (BPV) calculated of over the preceding 10 years (BPV period). Hazard ratios indicate lifetime risks and hazard ratios for mortality are independent of dementia because dementia is not a competing risk for mortality. Adjusted for sex, mean systolic blood pressure, years of education, smoking status, ApoE genotype and history of stroke, myocardial infarction and diabetes mellitus. Abbreviations: HR= hazard ratio, CI=confidence interval, BPV=blood pressure variability, SBP: systolic blood pressure

© 2023 den Brok MGHE et al. *JAMA Network Open*

eTable 4. Hazard ratios per one standard deviation increment in systolic BPV at ages 60, 70, 80, and 90 for incident dementia according to moment of censoring (<5 or ≥5 years of follow-up).

		< 5 years of follow-up		≥ 5 years of follow-up	
**Age**	**BPV period**	**Total / dementia events (%)**	**HR dementia (95%CI)**	**Total / dementia events (%)**	**HR dementia (95%CI)**
**60**	**50-59**	478 / 0 (0.00)	NA (NA - NA)	478 / 221 (46.2)	0.97 (0.85 - 1.11)
**70**	**60-69**	657 / 11 (1.7)	1.18 (0.50 - 2.78)	645 / 283 (43.9)	0.91 (0.81 - 1.03)
**80**	**70-79**	619 / 88 (14.2)	1.05 (0.83 - 1.32)	515 / 205 (39.8)	1.00 (0.87 - 1.16)
**90**	**80-89**	268 / 64 (23.9)	1.33 (0.96 - 1.85)	119 / 31 (26.1)	1.44 (0.91 - 2.25)

Cox proportional hazards for individuals at ages 60, 70, 80 and 90 years, alive without dementia, according to blood pressure variability (BPV) calculated of over the preceding 10 years (BPV period). Hazard ratios indicate lifetime risks. Adjusted for sex, mean systolic blood pressure, years of education, smoking status, ApoE genotype and history of stroke, myocardial infarction and diabetes mellitus. Note that HR for dementia<5 years was missing at age 60, because none of the participants developed dementia <5 years at that age. Abbreviations: HR= hazard ratio, CI=confidence interval, BPV=blood pressure variability, SBP: systolic blood pressure

© 2023 den Brok MGHE et al. *JAMA Network Open*

eTable 5. Hazard ratios per one standard deviation increment in systolic BPV at ages 60, 70, 80, and 90 for incident dementia according median to time to dementia diagnosis

			< median time to dementia		> median time to dementia	
**Age**	**BPV period**	**Median time to dementia, y**	**Total / dementia events (%)**	**HR dementia (95%CI)**	**Total / dementia events (%)**	**HR dementia (95%CI)**
**60**	**50-59**	26.0	478 / 108 (22.6)	1.06 (0.89 - 1.28)	281 / 113 (40.2)	0.90 (0.74 - 1.10)
**70**	**60-69**	16.5	657 / 151 (23.0)	0.91 (0.76 - 1.09)	371 / 139 (37.5)	0.89 (0.75 - 1.07)
**80**	**70-79**	8.5	619 / 154 (24.9)	1.03 (0.86 - 1.24)	351 / 117 (33.3)	0.99 (0.83 - 1.19)
**90**	**80-89**	3.5	268 / 44 (16.4)	1.15 (0.79 - 1.68)	156 / 41 (26.3)	1.48 (1.03 - 2.11)

Cox proportional hazards for individuals at ages 60, 70, 80 and 90 years, alive without dementia, according to blood pressure variability (BPV) calculated of over the preceding 10 years (BPV period). Hazard ratios indicate lifetime risks. Adjusted for sex, mean systolic blood pressure, years of education, smoking status, ApoE genotype and history of stroke, myocardial infarction and diabetes mellitus. Abbreviations: HR= hazard ratio, CI=confidence interval, BPV=blood pressure variability, SBP: systolic blood pressure

© 2023 den Brok MGHE et al. *JAMA Network Open*

eTable 6. Hazard ratios per one standard deviation increment in systolic BPV at ages 60, 70, 80, and 90 for incident dementia, excluding individuals with stroke at any age during life

Age	BPV period	Total / dementia events (%)	Total / mortality events (%)	HR dementia (95%CI)	HR mortality (95%CI)	HR dementia/ mortality (95%CI)
**60**	**50-59**	221 / 90 (40.7)	221 / 221 (100)	1.19 (0.93 - 1.53)	1.02 (0.87 - 1.19)	1.07 (0.91 - 1.25)
**70**	**60-69**	318 / 121 (38.1)	318 / 318 (100)	0.93 (0.76 - 1.13)	0.97 (0.87 - 1.10)	0.94 (0.84 - 1.06)
**80**	**70-79**	300 / 119 (39.7)	320 / 320 (100)	1.01 (0.83 - 1.23)	1.13 (1.00 - 1.28)	1.09 (0.96 - 1.23)
**90**	**80-89**	132 / 43 (32.6)	172 / 172 (100)	1.71 (1.10 - 2.64)	1.26 (1.02 - 1.57)	1.46 (1.12 - 1.90)

Cox proportional hazards for individuals at ages 60, 70, 80 and 90 years, alive without dementia, according to blood pressure variability (BPV) calculated of over the preceding 10 years (BPV period). Hazard ratios indicate lifetime risks and hazard ratios for mortality are independent of dementia because dementia is not a competing risk for mortality. Adjusted for sex, mean systolic blood pressure, years of education, smoking status, ApoE genotype and history of stroke, myocardial infarction and diabetes mellitus. Abbreviations: HR= hazard ratio, CI=confidence interval, BPV=blood pressure variability, SBP: systolic blood pressure

© 2023 den Brok MGHE et al. *JAMA Network Open*

eTable 7. Association for one SD increase in mean systolic blood pressure variability over previous 10-years and lifetime dementia or mortality risk, <u>excluding individuals with myocardial infarction at any age during life</u>

Age	BPV period	Total / dementia events (%)	Total / mortality events (%)	HR dementia (95%CI)	HR mortality (95%CI)	HR dementia/ mortality (95%CI)
**60**	**50-59**	261 / 119 (45.6)	261 / 261 (100)	1.01 (0.84 - 1.22)	1.06 (0.94 - 1.20)	1.03 (0.91 - 1.17)
**70**	**60-69**	353 / 158 (44.8)	353 / 353 (100)	1.00 (0.85 - 1.18)	1.01 (0.91 - 1.12)	0.98 (0.88 - 1.09)
**80**	**70-79**	323 / 142 (44.0)	344 / 344 (100)	1.08 (0.90 - 1.3)	1.13 (1.00 - 1.29)	1.15 (1.02 - 1.29)
**90**	**80-89**	132 / 42 (31.8)	189 / 189 (100)	1.48 (1.01 - 2.17)	1.19 (1.00- 1.41)	1.24 (0.98 - 1.55)

Cox proportional hazards for individuals at ages 60, 70, 80 and 90 years, alive without dementia, according to blood pressure variability (BPV) calculated of over the preceding 10 years (BPV period). Hazard ratios indicate lifetime risks and hazard ratios for mortality are independent of dementia because dementia is not a competing risk for mortality. Adjusted for sex, mean systolic blood pressure, years of education, smoking status, *APOE* genotype and history of stroke, myocardial infarction and diabetes mellitus. Abbreviations: HR= hazard ratio, CI=confidence interval, BPV=blood pressure variability, SBP: systolic blood pressure

© 2023 den Brok MGHE et al. *JAMA Network Open*

eTable 8. Association for one SD increase in mean systolic blood pressure variability over previous 10-years and lifetime dementia or mortality risk, using inverse probability weighting to account for potential selection into the autopsy cohort from the larger ACT study population

Age	BPV period	Total / dementia events (%)	Total / mortality events (%)	HR dementia (95%CI)	HR mortality (95%CI)	HR dementia/ mortality (95%CI)
**60**	**50-59**	484 / 220 (45.5)	484 / 484 (100)	1.00 (0.88 - 1.15)	1.04 (0.94 - 1.14)	1.03 (0.94 - 1.13)
**70**	**60-69**	657 / 290 (44.1)	658 / 658 (100)	0.90 (0.8 - 1.02)	0.99 (0.92 - 1.07)	0.95 (0.88 - 1.03)
**80**	**70-79**	617 / 273 (44.2)	659 / 659 (100)	0.98 (0.86 - 1.12)	**1.12 (1.03 - 1.22)**	1.08 (0.99 - 1.17)
**90**	**80-89**	265 / 84 (31.7)	374 / 374 (100)	1.29 (0.96 - 1.73)	**1.14 (1.00 - 1.29)**	**1.19 (1.00 - 1.42)**

Cox proportional hazards for individuals at ages 60, 70, 80 and 90 years, alive without dementia, according to blood pressure variability (BPV) calculated of over the preceding 10 years (BPV period). Hazard ratios indicate lifetime risks. Adjusted for sex, mean systolic blood pressure, years of education, smoking status, ApoE genotype and history of stroke, myocardial infarction and diabetes mellitus. Estimates and 95% confidence intervals were calculated using 10,000 bootstraps. See eMethods 2 for full description of the inverse probability weighting procedure and analyses. Abbreviations: HR= hazard ratio, CI=confidence interval, BPV=blood pressure variability, SBP: systolic blood pressure

© 2023 den Brok MGHE et al. *JAMA Network Open*

eTable 9. Association for one SD increase in mean systolic blood pressure variability over previous 10-years and lifetime dementia or mortality risk, only including individuals who had attended ACT baseline at or before the index age

Age	BPV period	Total / dementia events (%)	Total / mortality events (%)	HR dementia (95%CI)	HR mortality (95%CI)	HR dementia/ mortality (95%CI)
**60**	**50-59**	-	-	-	-	-
**70**	**60-69**	118 / 43 (36.4)	134 / 134 (100)	0.75 (0.51 - 1.10)	0.95 (0.79 - 1.14)	0.95 (0.79 - 1.14)
**80**	**70-79**	133 / 46 (34.6)	440 / 440 (100)	0.95 (0.8 - 1.13)	1.13 (1.01 - 1.26)	1.06 (0.95 - 1.19)
**90**	**80-89**	400 / 176 (44.0)	351 / 351 (100)	1.34 (1.00 - 1.81)	1.14 (1.00 - 1.29)	1.15 (0.97 - 1.37)

Cox proportional hazards for individuals at ages 60, 70, 80 and 90 years, alive without dementia, according to blood pressure variability (BPV) calculated of over the preceding 10 years (BPV period). Hazard ratios indicate lifetime risks. Adjusted for sex, mean systolic blood pressure, years of education, smoking status, ApoE genotype and history of stroke, myocardial infarction and diabetes mellitus. In these analyses, only individuals who were at risk from the index age (i.e. had had their ACT baseline measurement) were included in the analyses. Analyses for age 60 could not be performed because individuals were only included in the ACT from age 65 and older. Abbreviations: HR= hazard ratio, CI=confidence interval, BPV=blood pressure variability, SBP: systolic blood pressure

© 2023 den Brok MGHE et al. *JAMA Network Open*

eTable 10. Association for one SD increase in mean systolic blood pressure variability over previous 10-years and lifetime dementia or mortality risk, using 10-year BPV calculated with all available SBP measurements per individual per year (maximum 3) adjusted for the number of SBP measurements

Age	BPV period	Total / dementia events (%)	Total / mortality events (%)	HR dementia (95%CI)	HR mortality (95%CI)	HR dementia/ mortality (95%CI)
**60**	**50-59**	492 / 223 (45.3)	492 / 492 (100)	1.00 (0.88 - 1.14)	1.03 (0.94 - 1.13)	1.03 (0.94 - 1.13)
**70**	**60-69**	668 / 294 (44)	669 / 669 (100)	0.90 (0.8 - 1.02)	0.99 (0.91 - 1.07)	0.95 (0.88 - 1.03)
**80**	**70-79**	628 / 277 (44.1)	671 / 671 (100)	0.99 (0.87 - 1.13)	1.12 (1.03 - 1.22)	1.08 (0.99 - 1.17)
**90**	**80-89**	272 / 86 (31.6)	381 / 381 (100)	1.28 (0.96 - 1.72)	1.14 (1.00 - 1.3)	1.19 (1.01 - 1.42)

Cox proportional hazards for individuals at ages 60, 70, 80 and 90 years, alive without dementia, according to blood pressure variability (BPV) calculated of over the preceding 10 years (BPV period). Hazard ratios indicate lifetime risks. Adjusted for sex, mean systolic blood pressure, years of education, smoking status, ApoE genotype and history of stroke, myocardial infarction and diabetes mellitus. Abbreviations: HR= hazard ratio, CI=confidence interval, BPV=blood pressure variability, SBP: systolic blood pressure

eFigure. Depiction of BPV periods in relation to analysis index age.

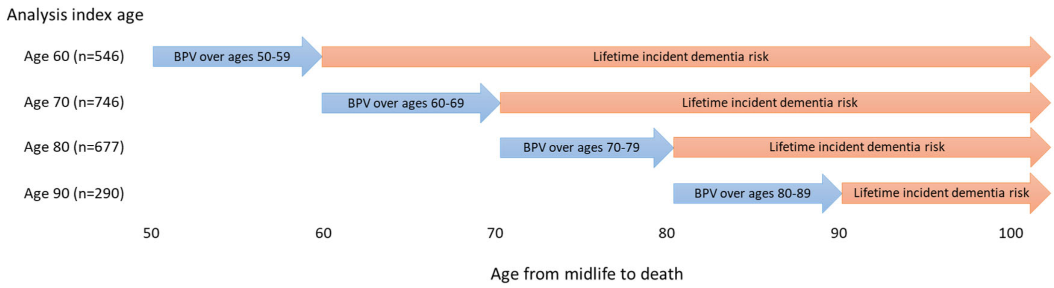

In blue the periods over which blood pressure variability (BPV) was measured and in pink the remaining lifetime over which dementia risk was analyzed. The number of individuals available per age stratum varies, because not all persons had data available from as early as age 50, and attrition due to death or dementia occurred before reaching the higher age strata.

© 2023 den Brok MGHE et al. *JAMA Network Open*
