# Supplementary material for: Year-by-Year Blood Pressure Variability From Midlife to Death and Lifetime Dementia Risk
Source: JAMA Netw Open. 2023 Oct 30;6(10):e2340249. doi: 10.1001/jamanetworkopen.2023.40249 (PMC10616718; doi:10.1001/jamanetworkopen.2023.40249)
Supplement: Supplement 2. — Data Sharing Statement [file jamanetwopen-e2340249-s002.pdf]

Data Sharing Statement

Data
Data available: Yes
Data types: Deidentified participant data
How to access data: The datasets used and/or analyzed in the current study are available from kpwa.actproposals@kp.org on reasonable request and execution of appropriate human subjects review and data sharing agreements.
When available: With publication

Supporting Documents
Document types: None

Additional Information
Who can access the data: Anyone requesting the data whose proposed use has been approved
Types of analyses: Any puropose upon reasonable request
Mechanisms of data availability: After approval of proposal
Any additional restrictions: The datasets used and/or analyzed in the current study are available from kpwa.actproposals@kp.org on reasonable request and execution of appropriate human subjects review and data sharing agreements.
